# Spatio-temporal expression and distribution of collagen VI during zebrafish development

**DOI:** 10.1038/s41598-019-56445-4

**Published:** 2019-12-27

**Authors:** Valentina Tonelotto, Valeria Trapani, Sandrine Bretaud, Stefanie Elisabeth Heumüller, Raimund Wagener, Florence Ruggiero, Paolo Bonaldo

**Affiliations:** 1 Department of Molecular Medicine, University of Padova, Padova, 35131 Italy; 2 Institut de Génomique Fonctionnelle de Lyon, ENS de Lyon, UMR CNRS 5242, Université Lyon 1, Lyon, 69364 France; 3 Center for Biochemistry and Center for Molecular Medicine Cologne (CMMC), Medical Faculty, University of Cologne, Cologne, 50931 Germany; 4 CRIBI Biotechnology Center, University of Padova, Padova, 35131 Italy

**Keywords:** Embryology, Zebrafish

## Abstract

Collagen VI (ColVI) is an extracellular matrix (ECM) protein involved in a range of physiological and pathological conditions. Zebrafish (*Danio rerio*) is a powerful model organism for studying vertebrate development and for *in vivo* analysis of tissue patterning. Here, we performed a thorough characterization of ColVI gene and protein expression in zebrafish during development and adult life. Bioinformatics analyses confirmed that zebrafish genome contains single genes encoding for α1(VI), α2(VI) and α3(VI) ColVI chains and duplicated genes encoding for α4(VI) chains. At 1 day post-fertilization (dpf) ColVI transcripts are expressed in myotomes, pectoral fin buds and developing epidermis, while from 2 dpf abundant transcript levels are present in myosepta, pectoral fins, axial vasculature, gut and craniofacial cartilage elements. Using newly generated polyclonal antibodies against zebrafish α1(VI) protein, we found that ColVI deposition in adult fish delineates distinct domains in the ECM of several organs, including cartilage, eye, skin, spleen and skeletal muscle. Altogether, these data provide the first detailed characterization of ColVI expression and ECM deposition in zebrafish, thus paving the way for further functional studies in this species.

## Introduction

Collagen VI (ColVI) is a major extracellular matrix (ECM) protein forming a microfibrillar network in many tissues, including skeletal muscle, cartilage, tendon, lung, nervous system, adipose tissue, skin, eye, heart and vasculature^1^. ColVI is a distinctive, non-fibrillar member of the superfamily of collagens. The protein is made of three major chains, α1(VI), α2(VI) and α3(VI), which are respectively encoded by the *COL6A1*, *COL6A2* and *COL6A3* genes in tetrapods^2–5^. More recently, three other genes were identified, annotated as *COL6A4*, *COL6A5* and *COL6A6*, whose expression is restricted to specific tissues^6,7^. In humans, a large chromosome inversion broke the *COL6A4* gene into two separate non-processed pseudogenes^8^. The domain structure of the α4(VI), α5(VI) and α6(VI) chains and the shared homology with α3(VI) indicate that they represent alternative polypeptides for the assembly of ColVI subunits into α1–α2–αX triple-helical monomers (where αX is either α3, α4, α5 or α6), thus increasing the structural and functional versatility of this ECM component^7,9^. Of note, ColVI has a unique intracellular assembly process made of distinct steps, where three different chains associate intracellularly to form disulfide-bonded monomers, dimers and tetramers before secretion^10,11^. In the extracellular microenvironment, ColVI forms a network of beaded microfilaments which interact with a range of other ECM molecules, including fibronectin, collagen IV, decorin and perlecan^1^. Moreover, ColVI is able to bind several cell surface receptors, such as the α1β1, α2β1, α3β1, α10β1 and αvβ3 integrins, the chondroitin sulfate proteoglycan-4 (CSPG4, also known as NG2), and the anthrax toxin receptors 1 and 2 (ANTXR1/TEM8 and ANTXR2/CMG2)^12–14^.

Several human disorders have been linked to altered expression or mutations of the genes encoding ColVI chains. In particular, ColVI is critical for the proper structure and function of skeletal muscle, and inherited mutations of the *COL6A1-COL6A3* genes cause different forms of myopathies in humans, including Ullrich congenital muscular dystrophy (UCMD) and Bethlem myopathy (BM) (for a review, see^15^). A number of animal models have been generated for myopathies with a spectrum of phenotypes^16^. The generation of a *Col6a1* null mouse model provided a valuable tool for the dissection of the *in vivo* functions of ColVI^17^, thereby highlighting the relevance of this ECM component in regulating several key cellular pathways. Indeed, ColVI exerts different functions in the tissues where it is expressed, including protection from apoptosis and from oxidative damage, regulation of autophagy, promotion of tumor growth and progression, and maintenance of cell stemness^1^. ColVI knockout mice display a myopathic phenotype characterized by structural and functional defects in skeletal muscles^17^. In particular, ColVI-deficient myofibers undergo spontaneous apoptosis, with accumulation of dysfunctional mitochondria and altered organelles^18^. Further studies demonstrated that the persistence of abnormal organelles in ColVI knockout muscles is caused by defective regulation of the autophagic machinery^19^. Of note, the pathomolecular defects identified in *Col6a1* null mice were also confirmed in muscle biopsies and primary muscle cultures of UCMD and BM patients^19,20^. Treatment with cyclosporin A was able to rescue the latent mitochondrial dysfunction and myofiber apoptosis of both ColVI knockout mice and UCMD/BM patients^18,21^. Further work demonstrated that administration of spermidine, a non-toxic cationic polyamine, reactivates autophagy in a dose-dependent manner in ColVI knockout mice, leading to a significant amelioration of muscle defects^22^. More recently, a pilot clinical trial was carried out, showing the efficacy of a one-year low-protein diet in reactivating autophagy in skeletal muscle of patients affected by ColVI myopathies^23^.

*Danio rerio* (zebrafish) has been widely used for studies of vertebrate development and gene function. Indeed, thanks to its transparency and rapid development, zebrafish represents a powerful tool to visualize the expression pattern of a gene in the whole organism. Moreover, this animal model allows the dissection of different aspects associated with specific gene functions, providing valuable data for a better understanding of human development and disease mechanisms, such as for collagenopathies^24,25^. Although some studies on ColVI in zebrafish were carried out in the past few years^26–28^, only limited data are available concerning the expression and distribution of ColVI during fish development and adult life. In this work, we investigated the spatio-temporal expression pattern of ColVI genes and the distribution of ColVI protein in zebrafish embryos, larvae and adults. The data demonstrate that zebrafish ColVI genes exhibit features that are very similar to those reported for their mammalian orthologs^7,29^. By exploiting a new antibody we generated against zebrafish ColVI, we characterized the expression pattern of this distinctive ECM protein during zebrafish development and adult life. These data are of major relevance since they provide the basis for further functional studies in this animal model.

## Results

### Phylogenetic analysis of fish ColVI genes

**Figure 1:**
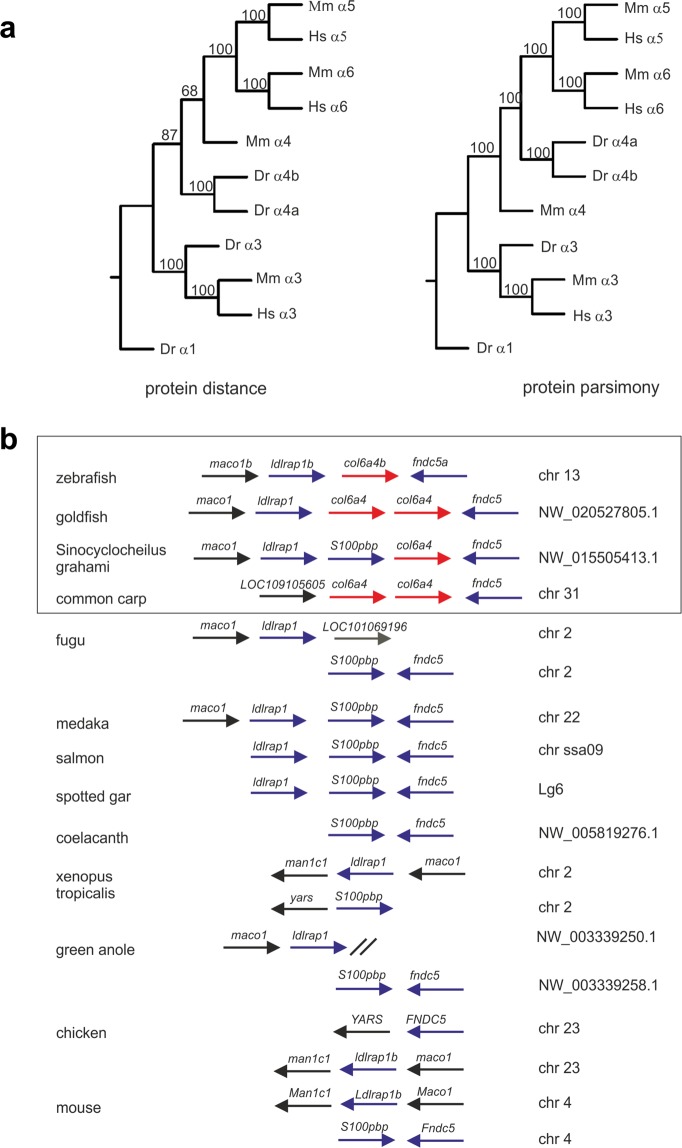
Phylogenetic analysis of zebrafish ColVI genes. (**a**) Phylogenetic trees of the ColVI α4, α5 and α6 chains in different species, obtained by amino acid sequence comparison of the regions spanning the C1 and C2 domains in the corresponding α chains. The sequences from zebrafish (z), mouse (m) and human (h) were aligned using the PILEUP program of the GCG package, using default parameters. The trees were constructed using the PROTEIN PARSIMONY, PROTEIN DISTANCE, FITCH and CONSENSE tools of the PHYLIP package version 3.69. Bootstrap analyses using 100 replicates were performed to show the significance. Numbers indicate the statistical weight of the individual branches. The C1 and C2 domains of zebrafish ColVI α1 chain were used as outgroup. (**b**) Comparative maps of syntenic regions of the zebrafish *col6a4b* gene. Genes encoding the corresponding α4 chain in different species are indicated by red arrows. Neighboring syntenic genes are indicated by blue arrows. For simplicity, the orientation of the genes on the chromosomes was adjusted to that of zebrafish chromosome 13. Members of the carp family (Cyprinidae) are boxed.

Five genes coding for ColVI chains were identified in zebrafish^25,27,30,31^, one ortholog each for the α1, α2 and α3 chains and two for the α4 chain (see also Supplementary Fig. S1). The two orthologs coding for the α4(VI) chain are designated *col6a4a* and *col6a4b* and are located on chromosome 16 and 13, respectively. However, it has not been determined how *col6a4a* and *col6a4b* evolved. Therefore, we performed a phylogenetic analysis based on protein parsimony and protein distance methods and confirmed earlier results^27^ indicating that the two zebrafish α4 chains do not belong to the α5/α6 chain branch (Fig. 1a and Supplementary Fig. S2a). The question remained whether the duplication of the two α4 genes is the result of the whole genome duplication^32^ that occurred in the teleost linage, or if these two genes were independently duplicated. Close inspection of the gene loci revealed that *col6a4a* (Supplementary Fig. S2b), but not *col6a4b* (Fig. 1b), is in synteny with the locus coding for the α4(VI) chain in other vertebrates. In amphibians, reptiles, birds and mammals, the genes *ldlrap1b* and *fndc5a* that flank *col6a4b* on zebrafish chromosome 13 lack a neighboring gene coding for a ColVI chain (Fig. 1b). Moreover, the synteny of orthologue *col6a4b* genes with *ldlrap1b* or *fndc5a* is only present in cyprinids (Cyprinidae) (Fig. 1b), also commonly called the “carp family”, clearly indicating that these ColVI α4 chains are unique for this family. The genomes of other teleosts, like salmon, fugu or medaka, and that of the spotted gar^33^, which belongs to the infraclass of holostei, do not contain a *col6a4b* gene (Fig. 1b). Consequently, in carps *col6a4a* and *col6a4b* are paralogue genes independently of the teleost whole genome duplication. Interestingly, in *Cyprinus carpio* (common carp) and *Carassius auratus* (goldfish), *col6a4b* is further duplicated in tandem (Fig. 1b). The existence of an extra ColVI chain unique for carps may point to a specific function of this chain in this family of fish.

### ColVI expression and distribution in developing zebrafish embryos and larvae

**Figure 2:**
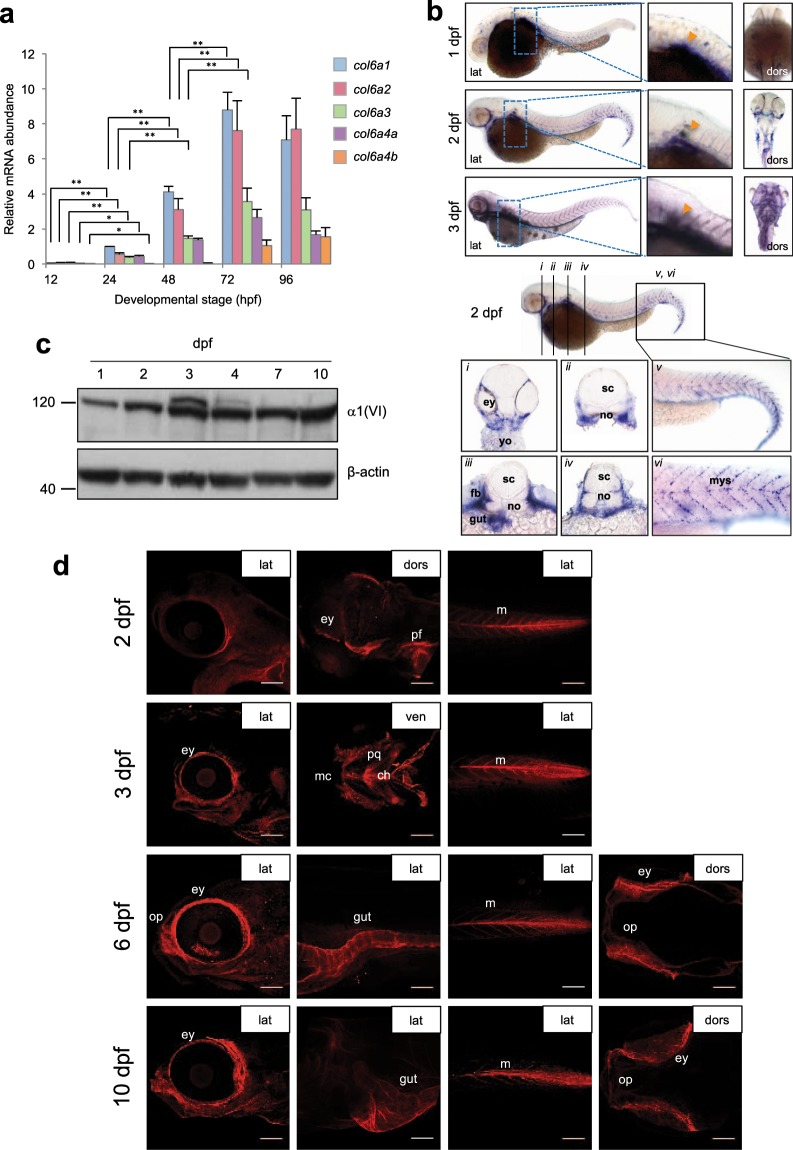
ColVI expression in zebrafish at different developmental stages. (**a**) qRT-PCR for *col6a1*, *col6a2*, *col6a3*, *col6a4a* and *col6a4b* transcripts in zebrafish embryos and larvae from 12 hpf to 96 hpf. Data were normalized to *arp*. The expression levels of the different genes were compared to *col6a1* expression at 24 hpf, which was arbitrarily set to 1, and represent the mean of at least three independent experiments. Error bars indicate s.e.m. (**P < 0.01). (**b**) Whole-mount *in situ* hybridization for *col6a1* in zebrafish embryos and larvae. The top panels show lateral (lat) and dorsal (dors) views of 1- to 3-dpf embryos labeled with the *col6a1* probe. The bottom panels show transverse sections at different head and trunk levels (*i-iv*, as indicated on the lateral view at the top) and higher magnifications of the tail region (*v* and *vi*) of 2-dpf embryos labeled with the *col6a1* probe. At 1 dpf, labeling for *col6a1* mRNA is restricted to two lines of slow muscle fibers and to the pectoral fin buds (orange arrowhead in the magnified area highlighted by the dotted box). At 2 and 3 dpf, strong signals for *col6a1* transcript are detected in vertical myosepta, pectoral fins (orange arrowhead in the magnified areas highlighted by the dotted boxes), branchial arches, axial vasculature and gut. All panels were cropped from the background. (**c**) Western blot for α1(VI) chain in protein extracts from zebrafish embryos and larvae at the indicated stages. β-actin was used as a loading control. Numbers on the left indicate sizes (in kDa) of protein standard markers. The extra-band observed at 3 and 4 pdf may correspond to post-translational modifications. The cropped blots for α1(VI) chain and β-actin derive from different parts of the same gel. (**d**) Whole-mount immunofluorescence labeling with anti-ColVI antibody in zebrafish larvae at different developmental stages from 2 dpf to 10 dpf, as indicated. The panels show lateral (lat), ventral (ven) and dorsal (dors) views of different head and trunk regions. From 2 dpf onwards, ColVI immunoreactivity (red) is present in the connective tissue surrounding the eye, in the pectoral fins and in myosepta. At 3 dpf, ColVI deposition extends to craniofacial elements and intestine. From 6 dpf onwards, ColVI labeling is also detected in the connective tissue surrounding olfactory pits. Scale bar, 100 µm. ch, ceratohyal; ey, eye; fb, fin buds; m, myosepta; mc, Meckel’s cartilage; myoseptum, mys; no, notochord; op, olfactory pit; pf, pectoral fins; pq, palatoquadrate; sc, spinal cord; yo, yolk.

To assess the expression of ColVI genes during fish development, we performed qRT-PCR at different developmental stages from 12 hpf to 4 dpf. ColVI transcripts were first detected at 1 dpf and then they increased throughout development. Notably, *col6a1* and *col6a2* mRNA levels were the most abundant at all developmental stages included in the analysis. Moreover, among the transcripts encoding the longer chains, the levels of *col6a3* and *col6a4b* mRNAs gradually increased between 1 dpf and 3 dpf, whereas *col6a4a* mRNA was barely detectable before 3 dpf (Fig. 2a).

To further investigate the spatio-temporal expression of ColVI transcripts during development, we performed *in situ* hybridization in zebrafish embryos and larvae from 1 dpf to 3 dpf, using digoxigenin-labeled antisense riboprobes for *col6a1*, *col6a2* and *col6a3*. Interestingly, at 1 dpf all three ColVI transcripts were restricted to the surface of the myotome and pectoral fin buds (Fig. 2b and Supplementary Fig. S3a). Of note, although ColVI is generally described as a dermal collagen, *col6a1* transcripts were clearly detected in the cuboid epithelial cells of the developing fish epidermis (Supplementary Fig. S3b). Moreover, at 2 dpf strong signals for ColVI transcripts were displayed by myosepta, pectoral fins, axial vasculature and the gut. Expression of ColVI genes encoding the three main chains was also found in the connective tissue surrounding the eye and in the craniofacial cartilage elements at 2 and 3 dpf (Fig. 2b and Supplementary Fig. S3a).

We next investigated the levels and distribution of ColVI protein during fish development. As available antibodies for human and murine ColVI did not provide adequate cross-reaction with the zebrafish protein, we generated specific antibodies for zebrafish ColVI (see also Materials and Methods). The specificity of these antibodies was validated by whole mount immunofluorescence of 2-dpf embryos injected with an exon skipping morpholino oligonucleotide targeting exons 9 of *col6a1*^26^. The resulting morphant embryos displayed markedly decreased staining at the level of vertical and horizontal myosepta (Supplementary Fig. S3c), a region in which ColVI is highly expressed in normal conditions (see below).

Western blot analysis with the newly generated anti-α1(VI) antibodies in protein extracts from 1- to 10-dpf embryos showed an abundant band at about 130 kDa, corresponding to the expected migration for the α1(VI) polypeptide. ColVI protein levels were already abundant at 1 dpf (Fig. 2c). Whole-mount immunofluorescence of embryos and larvae at different developmental stages from 2 to 10 dpf allowed to ascertain the distribution of ColVI protein during development. At 2 dpf, ColVI labeling was detected in the connective tissue surrounding the eye, in the pectoral fin and in the myosepta (Fig. 2d). A similar pattern of ColVI deposition was detected in 3-dpf larvae, which also displayed strong ColVI labeling in the developing craniofacial cartilages (Fig. 2d). In 6- and 10-dpf larvae, abundant ColVI labeling was present in the connective tissues of eyes and olfactory pits and in the gut (Fig. 2d). Double immunostaining with antibodies against collagen XII, a protein expressed in the connective tissue sheaths (fascia) that surrounds the tissues and organs of the body^34^, confirmed that ColVI signals derived from connective tissues, as revealed by the abundant co-localisation of these two proteins in 2-, 3- and 6-dpf larvae (Supplementary Fig. S4a).

**Figure 3:**
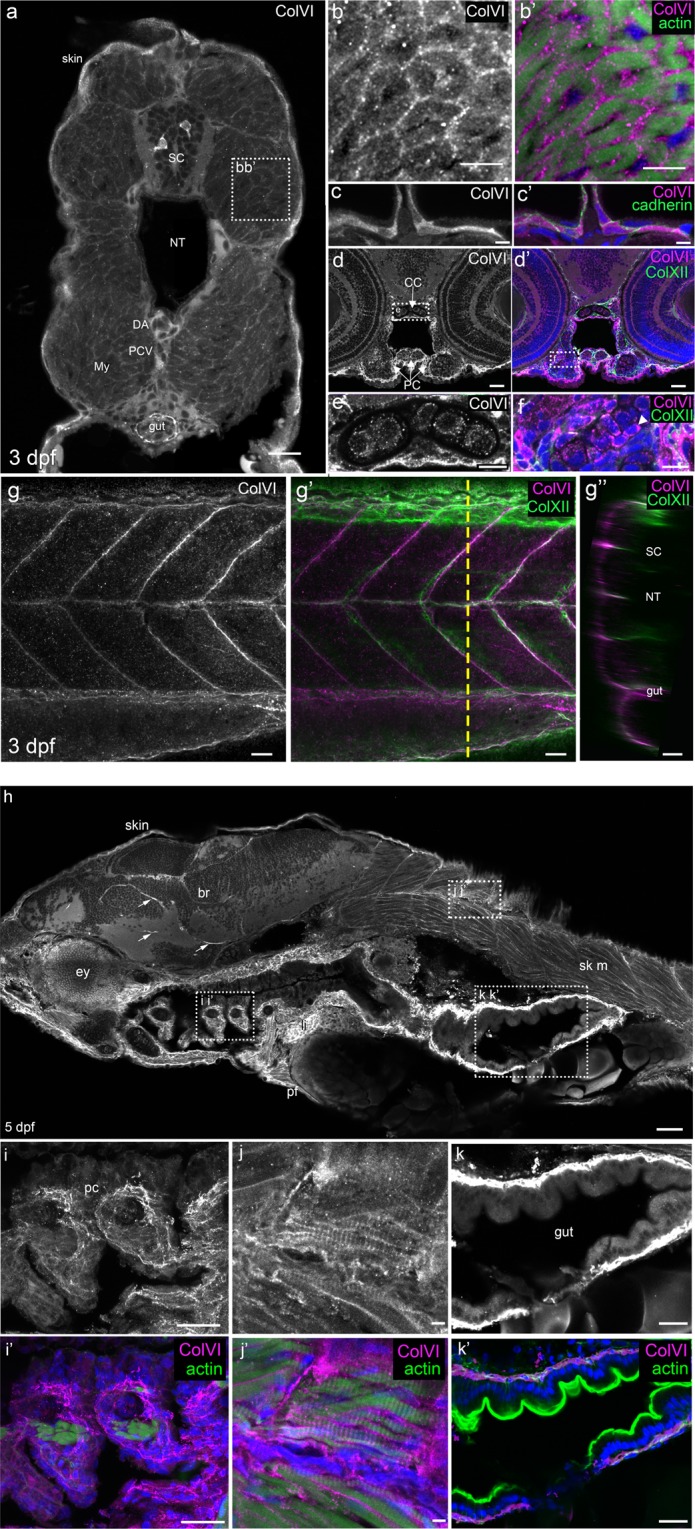
Characterization of ColVI deposition in 3- and 5-dpf developing zebrafish. (**a**-g’) Immunofluorescence images of transverse sections (**a**–**f**) or whole-mount (**g**-g”) 3-dpf larvae at the level of the trunk. Samples were labeled with anti-ColVI antibodies (panels a-e, g in grey; panels b’,c’,d’,f,g’,g” in magenta) and, where indicated, with phalloidin to reveal actin (green; panel b’), or with antibodies against the epithelial marker cadherin (green; panel c’) and the mesenchymal marker collagen XII (green; panels d’,f,g’,g”). Nuclei were stained with Hoechst (blue; panels b’,c’,d’,f). (**a**) Global view of ColVI immunoreactivity at the level of the trunk. (**b**,b’) Zoomed images of the boxed region in (**a**), showing myotomal muscle cells. (**c**,c’) Epidermis. (**d**,d’) Transverse section of the head. (**e**,**f**) Zoomed images of the boxed regions of panels d and d’, showing chondrocyte stacks in the cranial cartilage. White arrowhead points to intracellular ColVI staining in chondrocytes. (**g**) Image of vertical myosepta, obtained from a z-projection of confocal stack of lateral views. (**g’**) Merge image of the same acquisition, showing ColVI and collagen XII double staining. (g”) Orthogonal view at the level indicated by the yellow dashed line in g’. (**h**-k’) Immunofluorescence images of sagittal sections of 5-dpf larvae. Samples were labeled with α1(VI) antibodies (grey or magenta) and, where indicated, with phalloidin (green; panels i’,j’,k’). Nuclei were stained with Hoechst (blue; panels i’,j’,k’). (**h**) Global view of ColVI immunoreactivity. Arrows indicate ColVI-positive vessels. (**i**-**k’**) Zoomed images of the boxed regions of panel h. (**i**,**i’**) Ceratobranchial cartilage. (**j**,**j’**) Skeletal muscle at the trunk level. (**k**,k’) Intestine. Scale bars, 50 μm (**h**); 25 μm (**d**,d’,**g**-g”,**i**,i’,**k**,k’); 20 μm (**a**); 10 μm (**b**,b’,**e**,**f**); 5 μm (**c**,c’,**j**, j’). br, brain; CC, chondrocranium; DA, dorsal aorta; ey, eye; li, liver; My, myotome; NT, notochord; pc, pharyngeal cartilage; PCV, posterior cardinal vein; PC, pharyngeal cartilage; pf, pectoral fin; SC, spinal cord; sk m, skeletal muscle.

Further immunofluorescence analyses of whole-mount samples, as well as of frozen and paraffin-embedded sections, allowed to get additional insights on ColVI deposition during early development. At 3 dpf, transverse sections of the trunk (Fig. 3a–f) and whole-mount larvae (Fig. 3g,g’) showed ColVI deposition in skin, gut, skeletal muscles and vertical myosepta. Interestingly, orthogonal views of whole-mount larvae labelled with antibodies against collagen XII, a marker of myosepta^34^, revealed that ColVI was restricted to the surface of vertical myosepta (Fig. 3g”). This suggests a specific role of ColVI in the vertical myoseptum, a structure functionally equivalent to tetrapod tendon. Dorsal aorta and posterior cardinal vein were also positive for ColVI (Fig. 3a), but unstained controls showed autofluorescence of these structures (not shown), making it difficult to discern *bona fide* signal from autofluorescence. However, head vasculature did not show any autofluorescence and was strongly stained with ColVI antibodies (Supplementary Fig. S5a). Since *in situ* hybridization showed the presence of *col6a1* transcripts in various cell types (Fig. 2a and Supplementary Fig. S3b), we further examined ColVI protein deposition in various tissues and organs of 3 dpf larvae. Zoomed images of skeletal muscles co-stained for ColVI and actin revealed strong ColVI staining surrounding muscle fibers (Fig. 3b,b’). In agreement with our *in situ* hybridization data, strong ColVI immunoreactivity was found in skin (Fig. 3c). Co-immunostaining with antibodies to cadherin, a marker of epithelial cells, indicated that the basal cell layer of epidermis does produce ColVI (Fig. 3c’). In developing cartilage, ColVI immunoreactivity was found both in the perichondrium (Fig. 3d,d’) and chondrocytes (Fig. 3e,f).

Abundant ColVI deposition was displayed by various tissues of 5-dpf larvae, including head and trunk muscles, skin, liver, and the connective tissue surrounding the craniofacial cartilage elements (Fig. 3h). As mentioned above for 6-dpf larvae (Supplementary Fig. S5a), ColVI-positive blood vessels were present in the head (Fig. 3h). ColVI immunoreactivity in the connective tissue surrounding craniofacial cartilage elements and trunk muscles was clearly displayed by samples double stained with ColVI antibodies and phalloidin (Fig. 3i’,j’). Strikingly, ColVI immunoreactivity in muscle fibers alternated with actin staining, resulting in a costameric-like periodic pattern of ColVI deposition (Fig. 3j,j’). Finally, a strong ColVI labeling delineated the intestine (Fig. 3k), and co-staining with phalloidin indicated that ColVI is deposited in the smooth muscle layer of the developing intestine (Fig. 3k’).

**Figure 4:**
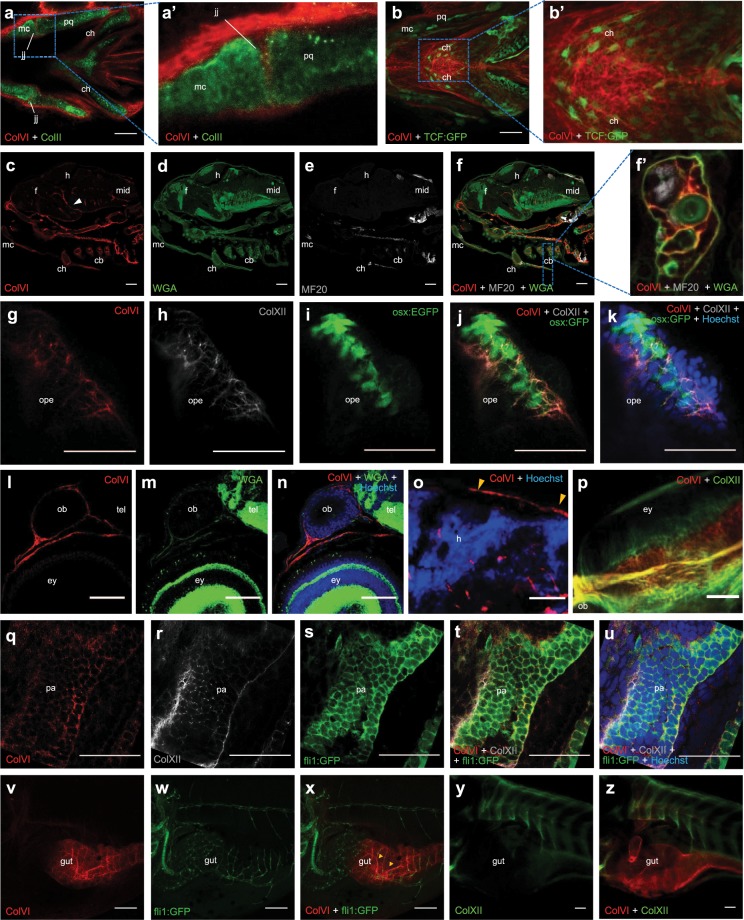
Spatio-temporal pattern of ColVI distribution in larvae from wild-type animals and transgenic reporter fish lines. (**a**-a’) Individual z-stack of whole-mount immunofluorescence for ColVI (red) and collagen II (green) in 3-dpf larvae. Ventral view. A strong ColVI labeling in evident in the connective tissue surrounding craniofacial cartilages and in the jaw joint. Panel **a’** is a magnification of the dotted area of panel **a**, showing the deposition of ColVI in the jaw joint. (**b**-b’) Confocal z-stacks of whole-mount immunofluorescence for ColVI (red) in 6-dpf *Tg(7xTCF-Xla*.*Siam:GFP)ia4* (TCF-GFP, green) larvae, showing ColVI labeling near Wnt-positive cells in craniofacial cartilages. Ventral view. Panel **b’** is a magnification of the dotted area of panel **b**, showing ColVI labeling in close apposition to Wnt-positive cells in the ceratohyal cartilages. (**c-**f’) Section of 7-dpf larvae stained with anti-α1(VI) antibodies (red), WGA (green) and anti-MF20 antibodies (gray). ColVI labeling is present in craniofacial cartilaginous elements, as well as in blood vessels (arrowhead). Panel **f’** is a magnification of the dotted area of panel f, showing ColVI labeling in the perichondrium surrounding the cartilaginous elements of the pharyngeal arches, as revealed by co-localization with WGA. (**g**–**k**) Individual z-stack of whole mount immunostaining of 6-dpf *Tg(osx:nuGFP)* (osx:GFP, green) larvae operculum, stained with ColVI (red) and collagen XII (gray) antibodies and Hoechst (blue). ColVI and collagen XII surround operculum osteoblasts. (**l**–**n**) Section of a 7-dpf larva stained with anti-ColVI antibodies (red), WGA (green) and Hoechst (blue). ColVI labeling is present in the connective tissue around brain, olfactory pit and eye. (**o**) Section of a 7-dpf larva stained with anti-ColVI antibodies (red) and Hoechst (blue), showing ColVI labeling in the meninges (arrowhead). (**p**) Confocal z-stacks of whole-mount immunofluorescence for ColVI (red) and collagen XII (green) on 6 dpf larvae eye, showing ColVI deposition in the connective tissue surrounding the eye. Dorsal view. (**q**–**u**) Individual z-stack of whole mount immunostaining for ColVI (red), collagen XII (gray) antibodies and Hoechst (blue) in 2-dpf *Tg(fli1:EGFP)* (fli:EGFP, green) larvae at the level of pharyngeal arches. ColVI and collagen XII are part of the bordering tissue of migrating pharyngeal pouches. (**v**–**x**) Confocal z-stacks of whole-mount immunofluorescence for ColVI (red) in 6-dpf *Tg(fli1:EGFP)* (fli:EGFP, green) larvae. A strong pattern of ColVI labeling is found around blood vessels (arrowheads) of the intestine. (**y-z**) Confocal z-stacks of whole-mount immunofluorescence for ColVI (red) and collagen XII (green) in 6-dpf larvae, showing that ColVI but not collagen XII is deposited in the intestine. Scale bar, 100 µm (**v**–**x**) or 50 µm (**a**–**u**,**y**,**z**). cb, ceratobranchial; ch, ceratohyal; ey, eye; f, forebrain; h, hindbrain; jj, jaw joint; mc, Meckel’s cartilage; mid, midbrain; op, olfactory pit; ope, operculum; pa, pharyngeal arch; pq, palatoquadrate.

In order to determine more precisely ColVI distribution at later developmental stages, we exploited different zebrafish transgenic reporter lines and antibodies for double staining analysis. First, we characterized ColVI deposition in the developing connective tissue of craniofacial structures by using an antibody against collagen II, a protein expressed in zebrafish craniofacial cartilages^35^. Analysis of 3-dpf larvae revealed that ColVI is expressed in the connective tissue surrounding Meckel’s, palatoquadrate and ceratohyals cartilages. Of note, ColVI deposition was found also in the jaw joint (Fig. 4a,a’; Supplementary Fig. S5b–e). Since one of the main molecular players involved in chondrogenesis is the Wnt signaling pathway, we also exploited *Tg(7xTCF-Xla*.*Siam:GFP)ia4*, a reporter fish line in which the expression of GFP is under the control of seven TCF responsive elements upstream the minimal promoter of *Xenopus* siamois gene, a direct β-catenin target^36^. At 3 and 6 dpf, ColVI was detected in close proximity to Wnt responsive cells at the level of ceratohyal cartilages (Fig. 4b,b’; Supplementary Fig. S5b–e) and at the level of the jaw joint (Fig. 4b,b’; Supplementary Fig. S5b–i). Of note, it was previously demonstrated that such Wnt-responding cells include chondrocytes of the jaw joint and along the palatoquadrate ligaments and tendons^37^. Immunofluorescence of 7-dpf sections confirmed ColVI deposition in branchial arches and chondrocranium (Fig. 4c-f’). In particular, co-staining with wheat germ agglutinin (WGA) and anti-light meromyosin (MF20) antibodies revealed that ColVI is localized in the perichondrium surrounding the cartilage of each pharyngeal arch, as well as in the connective tissue of the adjacent skeletal muscle (Fig. 4f’). To deepen the characterization of ColVI deposition in the developing craniofacial structures, we also took advantage of *Tg(osx:nuGFP)* reporter larvae, in which GFP expression mirrors endogenous *sp7* gene expression in the otic placode, and in the developing skeletal structures^38^. Analysis of 6-dpf *Tg(osx:nuGFP)* larvae revealed ColVI and collagen XII co-labeling in the operculum, a dermal bone which covers gills (Fig. 4g–k and Supplementary Fig. S5j–s). In the developing nervous system, ColVI was detected in brain blood vessels and in the meninges (Fig. 4l–o). Of note, in the connective tissue surrounding the eye ColVI partially co-localized with collagen XII (Fig. 4p). Moreover, using the *Tg(fli1:EGFP)* fish line, in which *fli1* promoter drives EGFP expression in cranial neural crest derivatives^39^, we detected ColVI deposition in the bordering tissue of migrating pharyngeal pouches (Fig. q–u and Supplementary Fig. S5t–x). As *fli1* promoter drives the expression of the EGFP in all blood vessels^39^, we found that ColVI labeling also followed the pattern of EGFP-positive cells at the level of the intestine, suggesting that ColVI is part of the connective tissue that underlies the epithelium of intestinal folds (Fig. 4v–x). Interestingly, ColVI, but not collagen XII, deposition was found in the intestine, suggesting that the two proteins do not co-localize in this organ (Fig. 4y–z).

### Characterization of ColVI expression and deposition in adult zebrafish tissues

**Figure 5:**
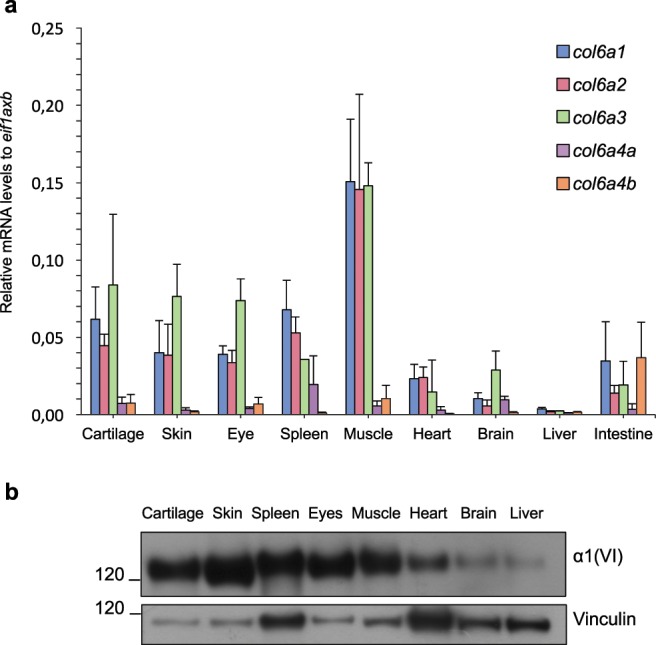
ColVI expression in adult zebrafish tissues. (**a**) qRT-PCR for *col6a1*, *col6a2*, *col6a3*, *col6a4a* and *col6a4b* transcripts in different tissues of adult (8- to 12-mpf) fish. Data were normalized to *eif1axb* expression. Error bars indicate s.e.m. (**b**) Western blotting for α1(VI) chain in protein extracts of adult (8- to 12-mpf) fish tissues. Vinculin was used as a loading control. Number on the left indicate sizes (in kDa) of protein standard markers. The cropped blots for α1(VI) chain and vinculin derive from different parts of the same gel.

To assess the levels of the ColVI transcripts during adulthood, we performed qRT-PCR on various tissues dissected from adult fish. High levels of the mRNAs coding for the three main ColVI chains were displayed by skeletal muscle, cartilage, skin, eye and spleen (Fig. 5a). Conversely, transcripts coding for the α4(VI) chain were expressed at much lower abundance in these tissues. Interestingly, high levels of the *col6a4* transcript were detected in the intestine (Fig. 5a), thus confirming previous results obtained for the murine α4(VI) chain^7^. Western blot analysis of protein extracts prepared from adult (8- to 12-mpf) animals showed that the α1(VI) chain distribution in the different tissues is comparable to the expression of *col6a1* transcripts.

**Figure 6:**
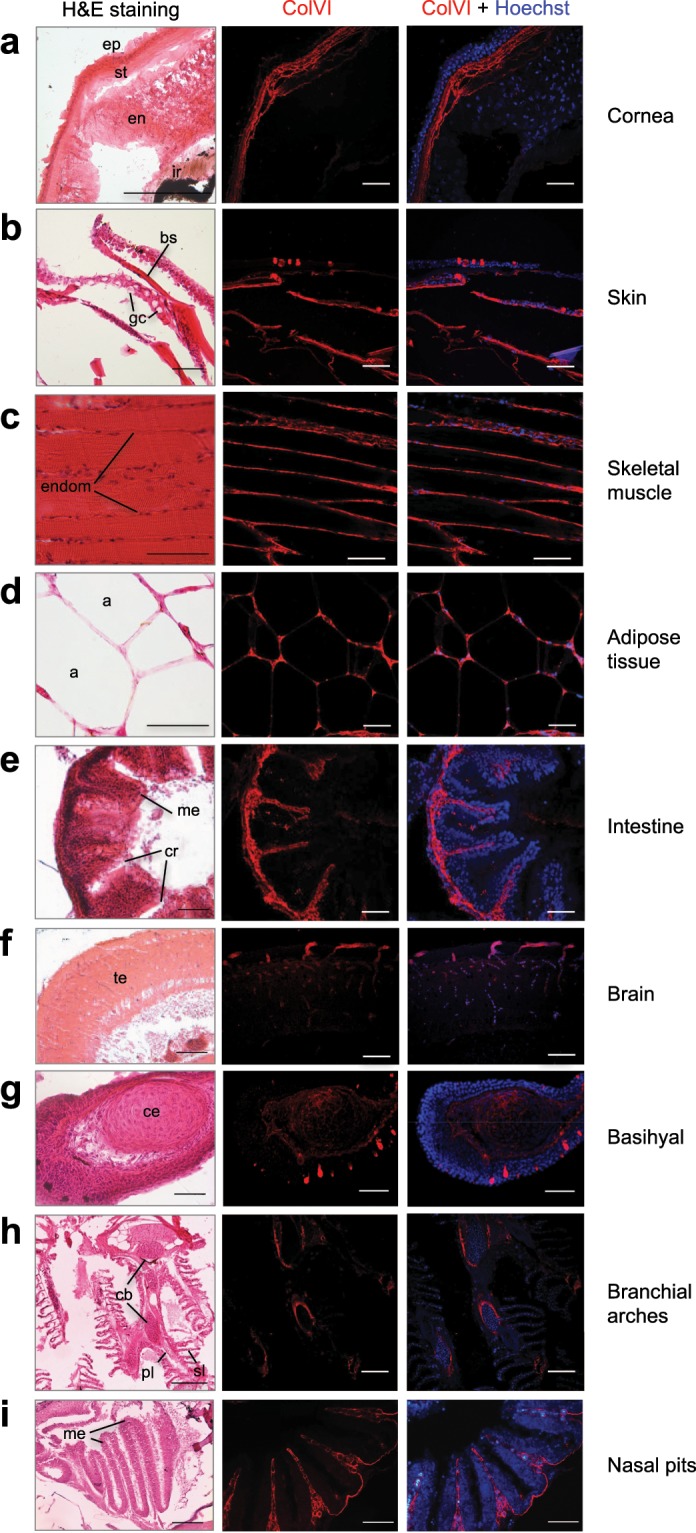
Characterization of ColVI deposition in adult zebrafish. (**a**–**i**) Sagittal sections of adult (8-to 12-mpf) fish tissues analyzed by haematoxylin-eosin staining (H&E, left panels) and by immunofluorescence labeling with anti-ColVI antibodies (red, middle and right panels). Nuclei were stained with Hoechst (blue, right panels). Scale bar, 50 µm. a, adipocyte; bs, bony scale; cb, ceratobranchial; ce, ceratohyal; cr, cryptae; endom, endomysium; en, endothelium; ep, epithelium; gc, goblet cell; ir, iris; me, mucosal epithelium, pl, primary lamella; sl, secondary lamella; st, stroma; te, telencephalon.

To further characterize the pattern of ColVI deposition in adult animals, we performed immunolabeling on sagittal sections of adult fish, followed by haematoxylin-eosin staining (Fig. 6a–i). ColVI immunoreactivity was abundant in the endomysium of skeletal muscle (Fig. 6c). In particular, the endomysial basement membrane was heavily stained, whereas the interstitial ECM displayed much lower ColVI labelling (Supplementary Fig. S6c). Cross sections of adult skin showed accumulation of ColVI filaments in the dermis (Fig. 5b and Supplementary Fig. S6a), indicating a cell switch of ColVI production in the skin at later stages, when fibroblasts are present in the dermis, as it was shown for collagen I^40^. Strong ColVI immunoreactivity was also displayed by the intestinal folds (Fig. 6e), in particular at the level of mucosa and muscular layers (Supplementary Fig. S6a). Of note, all these areas were shown to have abundant deposition of ColVI also in mice^7,29,41^. Moreover, ColVI signal was detected also in corneal stroma (Fig. 6a), in the adipose tissue (Fig. 6d), in brain blood vessels (Fig. 6f and Supplementary Fig. S6b–i), in the craniofacial cartilage elements (Fig. 6g,h; Supplementary Fig. S6j–q), and at the basal level of the olfactory epithelium (Fig. 6i). O note, in brain blood vessels and in the basihyal cartilage, ColVI was found in close apposition to Wnt-positive cells (Supplementary Fig. S6b–q). Interestingly, this analysis also revealed some fish-specific expression domains, such as bony scales and gill arches (Fig. 6b,h, respectively).

Taken together, these data demonstrate that, as in tetrapods, ColVI is broadly distributed in zebrafish during development and adult life, pointing at a role of this distinctive ECM component in the differentiation and specification of different tissues and organs.

## Discussion

Zebrafish is increasingly used as an animal model for investigating the function of collagen genes in development, regeneration and disease^25^. However, only partial data are currently available on the spatio-temporal expression patterns of the different collagens at the transcript and protein levels in zebrafish, in spite of the fact that such information is a fundamental prerequisite for undertaking functional studies in this animal model. In the present study, thanks to the generation of specific antibodies against zebrafish ColVI, we carried out a thorough characterization of the spatiotemporal expression and distribution of ColVI in zebrafish from early embryogenesis to adult life.

As in mammals, the fish genome contains several genes coding for distinct ColVI chains. Bioinformatics and phylogenetic analyses in zebrafish revealed the presence of single genes (*col6a1*, *col6a2* and *col6a3*) encoding the three major ColVI chains (α1, α2 and α3, respectively), and of two homologous genes (*col6a4a* and *col6a4b*) encoding α4 chains^26,27^ (see also Supplementary Fig. S1). These chains were earlier designated α4a and α4b^27^. However, the use of “a” and “b” is misleading in this context, as *col6a4a* and *col6a4b* are not ohnologs originating from the whole genome duplication that occurred in the teleost lineage. Ohnolog genes, generated by whole genome duplication and designated by “a” and “b” appendices, are common in zebrafish^42^. Only one of the two ohnolog genes, each encoding for ColVI chains, was maintained in zebrafish after the whole genome duplication. Nevertheless, a duplication of the *col6a4* gene occurred, but only in the carp family and not in the context of whole genome duplication. Interestingly, *col6a4* belongs to the subgroup of ColVI long-chain encoding genes^8^ that in very different species are independently duplicated (Supplementary Fig. S2b), indicating a susceptibility for duplication events. The basis for this susceptibility remains unclear. As the two zebrafish ColVI α4 chains are differentially expressed and the corresponding knockdown fish display different phenotypes^27^, caution is required when using results obtained from the study of zebrafish ColVI α4 chains to understand functions of α4(VI) in higher vertebrates, as one of the chains may have gained a novel function, unique for the carp family.

As for tetrapods, expression of ColVI genes in zebrafish is tighly regulated during embryonic and adult life. ColVI transcripts are first detected in 1-dpf fish embryos, and their expression increases throughout development. *In situ* hybridization of 1- to 3-dpf embryos showed the presence of *col6a1*, *col6a2* and *col6a3* transcripts in myotomes and developing craniofacial cartilages, as well as in eyes and gut. Interestingly, this expression pattern is comparable to that of the mammalian orthologs^7,29^, suggesting that the spatial and temporal regulation of ColVI genes expression is conserved among vertebrates. In particular, *col6a1* and *col6a2* transcript levels are the most abundant at all developmental stages, in agreement with the requirement of α1(VI) and α2(VI) for the 1:1:1 stoichiometric association with one of the longer chains. On the other hand, *col6a3* and *col6a4b* expression levels gradually increase between 1 and 3 dpf, whereas *col6a4a* levels increase between 3 and 4 dpf. This temporally regulated expression likely reflects different requirements of each of the longer ColVI chain in specific tissues during development. For instance, at 3 dpf, *col6a3* transcripts are abundant in pharyngeal cartilage elements, suggesting that the α3(VI) chain may be involved in craniofacial cartilage development, which typically occurs at this developmental stage^43^. In support of this notion, *col6a4b* expression also appears to be tissue-specific. Indeed, during larval stages *col6a4b* transcripts are first detected between 3 and 4 dpf, when gut differentiation is taking place^43^, and in adult animals *col6a4b* is mainly expressed in intestine.

Thanks to specific antibodies raised against the zebrafish α1(VI) chain, we were able to characterize ColVI protein distribution in both larvae and adults. In the developing zebrafish, ColVI is abundant in the anterior body region, including the connective tissues surrounding the eye, the brain and the olfactory epithelium, as well as in craniofacial cartilages and pectoral fins. In the trunk region, the protein is found in the intestine and in the myosepta. Immunostaining of zebrafish transgenic biosensor reporter lines allowed define more exhaustively the distribution of ColVI in different organs and tissues, showing ColVI deposition in the perichondrium surrounding Meckel’s and palatoquadrate cartilages and in the cartilaginous compartments of branchial arches. Interestingly, previous studies in mice showed that ColVI is abundant in the pericellular matrix of cartilage, where it is involved in the proper adhesion and function of chondrocytes^44^. ColVI is also present around the dentary bone and in the operculum of 6-dpf fish larvae. These results are in line with those reported in humans, where ColVI is found on the bone surface during development^45^, and also in mice, showing ColVI deposition in the mesenchymal region at the surface of skeletal elements^29^. One of the major molecular players involved in the control of vertebrate chondrogenesis is the Wnt signaling pathway^46^. Interestingly, in 6-dpf fish larvae ColVI displays abundant deposition in the jaw, near some clusters of cells expressing Wnt reporter activity. A recent study showed that Wnt responsive cells include chondrocytes at the jaw joint and along the palatoquadrate cartilage, as well as ligament and tendon fibroblasts^37^. These cells are located in areas subjected to high levels of tensile and compressive strain and include cell types known to respond to biomechanical stimuli, such as chondrocytes and tenocytes^37^. Based on these results, in the next future it will be interesting to carry out targeted functional studies in zebrafish, in order to ascertain if ColVI is required for cartilage development and maintenance and whether there is a link between this ECM protein and Wnt signaling in regulating such processes. Concerning tendons, our data also revealed strong ColVI reactivity in the myotome of zebrafish larvae. In particular, the protein is found at the level of vertical and horizontal myosepta, the teleost homolog of tendons. In mammals ColVI is localized in the pericellular region surrounding tendon fibroblasts^47^. Interestingly, previous studies showed that the tendons of *Col6a1*^*−/−*^ mice have disrupted microdomains and abnormal fibrillogenesis, with a decrease in maximum load and stiffness, thus indicating that ColVI contributes to the maintenance of the mechanical properties of tendons^47^.

Another tissue where ColVI is abundant in developing fish larvae is the mesenchyme surrounding brain, eyes and olfactory pits. At later stages, the protein is abundantly deposited in the corneal stroma and in the connective tissue that underlies olfactory epithelium. This distribution once again is in good agreement with what has been reported in mammals, where ColVI deposition was found in meningeal cells^29,48^ in the ECM of cornea^29,49^ and in the mesenchyme underneath the epithelium of nasal cavity^29^. Our immunofluorescence experiments also showed ColVI deposition in brain blood vessels both during development and in adulthood. In particular, ColVI is found in close apposition to Wnt-positive cells, previously reported as endothelial cells^29^. Given the fact that endothelial Wnt/β-catenin signaling regulates the induction and maintenance of blood-brain barrier features during embryonic and postnatal development^50,51^, it will be interesting to investigate whether ColVI plays a role in trapping and accumulating Wnt ligands, thus contributing to the fine regulation of Wnt/β-catenin signaling within the blood-brain barrier.

During adult life ColVI is broadly distributed in various tissues in mammals^1^, and other tissues showing ColVI labeling in zebrafish include intestine, adipose tissue, skin and skeletal muscles. Interestingly, we detected the protein in the connective tissue underlying the epithelium of intestinal folds not only in early larval stages but also in adult fish, as previously reported in mice^7,29^. Moreover, ColVI is abundant in the ECM surrounding adipocytes of adult fish, in agreement with the deposition pattern reported in humans^1^. In this respect, it has been demonstrated that adipocyte-derived ColVI promotes early mammary tumor progression by binding the NG2/CSPG4 cell surface receptor and triggering the activation of the Wnt/β-catenin pathway^52^. These data once again strongly suggest that the interaction between ColVI and Wnt signaling may play crucial roles in both physiological and pathological conditions. Concerning skin, in mammals ColVI is abundantly secreted by dermal fibroblasts, and *Col6a1* null mice represent a useful model for the study of skin pathology in patients affected by ColVI-related myopathies^53,54^. In zebrafish, *col6a1* transcripts are clearly detectable in the cuboid epithelial cells of developing epidermis. This is not surprising since the dermis remains acellular at early stages of skin development in zebrafish, and epithelial cells were found to be responsible for the deposition of collagen I and other ECM proteins in the dermis during development^40^. In adult fish, ColVI is strongly expressed in in the dermis, indicating a cell type switch of ColVI production in the skin at later stages, when fibroblasts are present in the dermis, as it was previously shown for collagen I^40^. In addition, adult fish displayed abundant ColVI deposition in bony scales, dermal derivatives involved in protection and hydrodynamics of swimming^55^. One of the main tissues showing ColVI expression in different species is skeletal muscle^1^. Interestingly, our data revealed a costameric-like periodic pattern of ColVI immunoreactivity in muscles of developing fish, suggesting that muscle fibers may contribute to ColVI deposition during early larval stages. In adult fish, ColVI is deposited in the endomysium and perimysium of muscle cells, where it is most likely produced by interstitial fibroblasts, as shown in mammals^7,15,56,57^. Of note, ColVI was recently found to be part of the specialized ECM of the neuromuscular junction (NMJ) of mice and humans, where it is required for the structural and functional integrity of this compartment^58^. Those studies revealed a novel role for ColVI in the NMJ, pointing at the involvement of NMJ alterations in patients affected by ColVI-related myopathies. Since NMJ development is very similar in zebrafish and humans^59^, it will be interesting to determine whether ColVI may have a critical role in regulating NMJ development. Indeed, the zebrafish model represents a valuable tool for such studies, as indicated by previous literature studies on the role of the ECM in motor axon pathfinding and neuromuscular development^59,60^.

Taken together, our results provide novel information on the expression and tissue-specific distribution of ColVI in zebrafish during development and adult life. In the next future, loss-of-function studies in this animal model will help dissecting the roles played by ColVI in various tissues, not only under physiological but also in pathological conditions. For this purpose, zebrafish represents an ideal model, given its rapid development and the availability of different transgenic biosensor lines. In this respect, our data represent a valuable basis for future work focused on the targeted inactivation of ColVI genes in zebrafish and aimed at shedding new light on the molecular mechanisms underlying the roles of this major ECM component in the modulation of signaling pathways and in tissue homeostasis during development and adulthood. The generation of such zebrafish models is particularly relevant, since they may help gaining further insight on the pathomolecular mechanisms that underlie ColVI-related diseases. Moreover, these zebrafish models will also represent suitable tools for future drug testing aimed at the urgent quest for efficacious treatments and therapeutic opportunities for genetic pathologies associated with ColVI deficiency.

## Materials and Methods

### Bioinformatics

For the analysis of gene loci, the NCBI genome data viewer (https://www.ncbi.nlm.nih.gov/genome/gdv/) was used. Multiple sequence alignments of the C1 and C2 domains of ColVI chains^1^ were performed using the Pileup algorithm of the Wisconsin Package^TM^. The phylogenetic analysis was done by protein distance and protein parsimony as described in PHYLIP v3.69.

### Animals

Maintenance and staging of AB/TU wild-type and transgenic [*Tg(osx:nuGFP)*, *Tg(fli1:EGFP)* and *Tg(7xTCF-Xla*.*Siam:GFP)ia4]* zebrafish were carried out using established protocols^43^. Fish were raised in a 14 h light cycle at approximately 28.5 °C. From 24 h post-fertilization (hpf), embryos were treated with phenylthiourea to block pigmentation. All animal manipulations were performed in agreement with EU Directive 2010/63/EU and were authorized by the University of Padova, Body for the Protection of Animals (OPBA-Project Number 1030/2015).

### Quantitative real-time PCR (qRT-PCR)

Organs were dissected from anesthetized adults. Total RNA of embryos, larvae (~30 for each experiment) and adult organs was extracted using TRIzol Reagent (Life Technologies), following the manufacturer’s protocol. One µg total RNA was retrotranscribed using the SuperScript III First-Strand Synthesis System for RT-PCR (Life Technologies), following manufacturer’s instructions. Resulting cDNAs were used to perform qRTPCR with Rotor-Gene SYBR Green PCR Kit mastermix (Qiagen) and a RotorGeneQ instrument (Qiagen). Primer sequences are shown in Table S1. Data on embryos and larvae were normalized to the *arp* housekeeping gene, coding for the arginine rich protein^61^, and the expression levels of the different genes were compared to *col6a1* expression at 1 dpf, which was arbitrarily set to 1. Data on adults were normalized to expression of the *eif1axb* housekeeping gene.

### Whole-mount *in situ* hybridization

Whole-mount *in situ* hybridization was performed following established protocols^62^. Digoxygenin uridine-5′-triphosphate-labelled RNA probes targeting the *col6a1*, *col6a2* and *col6a3* genes were generated. The primers used are listed in Table S2. Transverse sections (30 µm) were made with a Leica VT1000S vibratome. Stained embryos were analyzed under a Leica DMR compound/Nomarski microscope equipped with a Leica DC500 digital camera.

### Expression of recombinant zebrafish α1(VI) C-terminal globular domain and generation of specific antibodies

A cDNA construct encoding the C-terminal globular domains of the α(VI) chain was generated by PCR on total RNA from zebrafish larvae and cloned with 5′-terminal *Spe*I and 3′-terminal *Xho*I restriction sites, using the following primers: zfC6α1(f), 5′– gca act agt ATG CAC ATG TGG ACC CTT GGA –3′; zfC6α1(r), 5′– aac ctc gag CCC TCT CGT CTC CAG GGA AA –3′. The amplified PCR product was inserted into a modified pCEP-Pu vector containing an Nterminal BM-40 signal peptide and a C-terminal One-STrEP tag downstream the restriction sites^63^. Using FuGENE 6 transfection reagents (Roche), the recombinant plasmid was introduced into HEK293-EBNA cells (Invitrogen) according to manufacturer’s protocol. Selection was carried out with puromycin (1 µg/ml) and cells producing the One-STrEP-tagged protein were transferred to serum free medium for harvesting. Following filtration and centrifugation, the cell culture supernatants were applied to a Streptactin column (1.5 ml, IBA GmbH) and eluted with 2.5 mM desthiobiotin, 10 mM Tris-HCl, pH 8.0. Purified recombinant α1(VI) C-terminal polypeptide was used for rabbit immunization, and the obtained antiserum was purified by affinity chromatography on a column with the antigen coupled to CNBr-activated Sepharose^TM^ 4B (GE Healthcare Life Sciences). Bound antibodies were eluted with 0.1 M glycine, pH 2.5, and neutralized with 3 M TrisHCl, pH 8.8 and 5 M NaCl. The specificity of purified antibodies was determined by ELISA binding assay and immunoblotting.

### Morpholinos and microinjections

Embryos were injected with an exon skipping morpholino oligonucleotide targeting exons 9 of *col6a1* as described in^26^.

### Immunofluorescence

Immunohistochemical stainings of whole zebrafish embryos and larvae at 2, 3, 6 and 10 dpf were performed by the One for All protocol^64^. Primary antibodies against zebrafish α1(VI) (1:50; except for Fig. 3g, 1:400; rabbit polyclonal), collagen II (1:100; II-II6B3, mouse monoclonal, DSHB**)**, collagen XII (1:250; guinea pig^34^); and GFP (1:400; ab13970, chicken polyclonal, Abcam) were used, followed by anti-rabbit Cy3/Cy5 (1:500; Jackson Immunoresearch), anti-mouse FITC (1:500; Life Technologies), anti-guinea pig Cy3 (1:500, Jackson Immunoresearch) and anti-chicken Alexa Fluor 488 (1:500; Abcam), secondary antibodies. Nuclei were stained with Hoechst 33258 (1.5 µg/ml; Sigma). Whole mount immunohistochemical staining of Fig. 3c was performed following the protocol in^34^. Anti- zebrafish α1(VI) (1:400) and anti-collagen XII (1:250; guinea pig**)** were used as primary antibodies. Conjugated secondary antirabbit Alexa546 and anti-guinea pig Alexa-488 antibodies (both from Life Sciences) were used at 1:500. Immunostaining of frozen tissue sections was performed as in^59^, using antibodies against zebrafish α1(VI) (1:400), collagen XII (1:250^34^), E-cadherin (1:200; mouse BD Biosciences) and phalloidin-TRITC (1:100; Sigma). The secondary antibodies used were anti-rabbit Alexa546, anti-mouse Alexa488 and anti-guinea pig Alexa-488 (1:500, all from Life Sciences). Nuclei were stained as above. For some experiments, immunostaining was performed on paraffinized sections of 7-dpf and adult (8- to 12-mpf) zebrafish, obtained as in^65^. After deparaffination and rehydration, slices were covered with 10 mM sodium citrate buffer (pH 6.0) and placed in a steamer for 25 min, to promote antigen unmasking. In order to decrease autofluorescence, 50 mM NH_4_Cl was then placed on each slice for 1 h. After washing with phosphate buffered saline (PBS), slices were incubated for 30 min with 5% goat/sheep serum in PBS. Primary antibodies were diluted in 5% goat/sheep serum and applied overnight at 4 °C. In other experiments (Fig. 5e,i), adult zebrafish were fixed overnight with 4% paraformaldehyde and then frozen in liquid nitrogen. Sagittal sections (50 µm) made with a cryostat were then permeabilized for 10 min in cold 50% methanol-50% acetone at −20 °C and dried. After washing with PBS, slices were incubated for 30 min with 5% goat/sheep serum in PBS. The primary antibodies were diluted in 5% goat/sheep serum and applied overnight at 4 °C. Primary antibodies against the following proteins were used: zebrafish α1(VI) (1:400); GFP (1:400; ab13970, chicken polyclonal, Abcam); light meromyosin (1:100; MF20, mouse monoclonal, DHS). After washing in PBS, sections were then incubated with secondary antibodies for 1 h at room temperature. The following secondary antibodies were used: anti-rabbit Cy3/Cy5 (1:500; Jackson Immunoresearch); anti-mouse FITC (1:500; Life Technologies); anti-chicken Alexa Fluor 488 (1:500; Abcam). After brief washes, slices were incubated with Hoechst 33258 (see above) and mounted using 50 to 80% glycerol. Images were acquired with Leica SP5, Nikon C2 or Zeiss LSM 700 confocal microscopes and analyzed with the ImageJ/Fiji software.

### Histology

Adult zebrafish and 7-dpf larvae were fixed for 24 h in Bouin’s solution, following established protocols^65^. The samples were dehydrated progressively in ethanol and embedded in paraffin. Sagittal sections (7 µm) were made on Jung AG Heidelberg microtome. Slices were then deparaffinized, rehydrated and stained with haematoxylin and eosin. Finally, sections were mounted with Eukitt (BioOptica) for microscopic examination. In most cases, haematoxylin-eosin staining was performed on the same slices used for immunohistochemical experiments. Images were captured using a 5000B Leica microscope equipped with a DC500 digital camera.

### Western blotting

Embryos and larvae were deyolked (except for 7- and 10-dpf larvae) and lysed using Tissue Extraction Reagent I (Invitrogen) and proteases inhibitors (Complete EDTA free, Roche). Lysates were then processed through mechanical homogenization and protein concentration determined by the BCA Protein Assay kit (Pierce). Adult tissues were dissected following established protocols^66^, and lysis performed in Laemmli sample buffer containing 2 M urea and 50 mM dithiothreitol. SDS-PAGE of protein lysates (40–50 µg) was carried out in 3–8% polyacrylamide Novex NuPAGE Bis-Tris gels (Invitrogen), followed by electrotransfer onto PDVF membrane (Millipore). Membranes were blocked for 1 h in 5% milk in Tris-buffered saline/0.1% Tween 20 (TBST) and incubated overnight at 4 °C with antibodies against zebrafish α1(VI) (1:500) or β-actin (1:1000; mouse monoclonal, Sigma-Aldrich). Membranes were then washed three times with TBST and incubated for 1 h at room temperature with HRP-conjugated anti-rabbit or antimouse antibodies (1:1000; Amersham Bioscience). Detection was performed by SuperSignal West Pico or Dura Chemiluminescent Substrate with CL-XPosure Film (Thermo Scientific).

### Statistics

Data were analyzed through GraphPad Prism software. Mann-Whitney tests were used for all pairwise comparisons. Values were expressed as mean ± standard error.

## Supplementary information

Supplementary information

## Data Availability

The datasets generated and analysed during the current study are available from the corresponding author on reasonable request.
