# Supplementary material for: Spatio-temporal expression and distribution of collagen VI during zebrafish development
Source: Sci Rep. 2019 Dec 27;9:19851. doi: 10.1038/s41598-019-56445-4 (PMC6934817; doi:10.1038/s41598-019-56445-4)
Supplement: Supplementary file 1 — Supplementary information [file 41598_2019_56445_MOESM1_ESM.pdf]

Spatio-temporal expression and distribution of collagen VI during zebrafish development

Valentina Tonelotto[1, †], Valeria Trapani[1, †], Sandrine Bretaud[2], Stefanie Elisabeth Heumüller[3], Raimund Wagener[3], Florence Ruggiero[2, *] and Paolo Bonaldo[1, 4, *]

[1] Department of Molecular Medicine, University of Padova, Padova 35131, Italy.

[2] Institut de Génomique Fonctionnelle de Lyon, ENS de Lyon, UMR CNRS 5242, Université Lyon 1, Lyon 69364, France.

[3] Center for Biochemistry and Center for Molecular Medicine Cologne (CMMC), Medical Faculty, University of Cologne, Cologne 50931, Germany.

[4] CRIBI Biotechnology Center, University of Padova, Padova 35131, Italy.

[†] These authors contributed equally to this work.

Authors for correspondence: Paolo Bonaldo, Department of Molecular Medicine, University of Padova, Via Ugo Bassi 58/B, 35131 Padova, Italy, email: bonaldo@bio.unipd.it; Florence Ruggiero, Institut de Génomique Fonctionnelle de Lyon, ENS de Lyon, UMR CNRS 5242, Université Lyon 1, 69364 Lyon Cedex 07, France, email: florence.ruggiero@ens-lyon.fr

SUPPLEMENTARY MATERIAL

SUPPLEMENTARY TABLES

Supplementary Table 1. List of the primer sequences used for qRT-PCR. Ensembl gene names and ID numbers are underlined.

**Primer**	**Forward** (5’ -> 3’)	**Reverse** (5’ -> 3’)
qPCRzf*col6a1*(1)<br>col6a1-201 ENSDART00000110608.4	CATCAAGACCCTGACCGACC	GCACAGCACGTTTAATGGCA
qPCRzf*col6a1*(2)	CAAACGCTCTCAACTTCGCC	TGCTCCACGGCTTTCTCAAT
qPCRzf*col6a2*(1)<br>col6a2-201 ENSDART00000087597.6	CGGTGCCCTGGATATTGTGT	GTTGAGTCGAATGGCCTGGA
qPCRzf*col6a2*(2)	CTGTGCTTTGTCCGGATCCT	AAGACCAGATCAACAGGCCG
qPCRzf*col6a3*(1)<br>col6a3 ENSDARG00000077139	CATCCTCGCCACTTGAAGGT	CCAACCGCCATCACCACTAT
qPCRzf*col6a3*(2)	GGACTTCTGTGCCCCTGATC	TGGTGGGATCTCTTCGGTCT
qPCRzf*col6a4a*(1)	GCGCCGACTTTGAAGATGTG	TGAAGAGTGGGATTGCGGTC
qPCRzf*col6a4a*(2)	CACTTCAAATCGCCGCAACA	TCCTCAGAGCAATGACCCCA
qPCRzf*col6a4b*(1)	AACAGGGACATAGGGCAAGC	AGCCTCGATTCACTCTCCGA
qPCRzf*col6a4b*(2)	CGGAGAGTGAATCGAGGCTC	AACCTGCATTGCTCGTCTCA
qPCRzf*ef1axb*<br>eef1a1l1 ENSDARG00000020850	CTGGAGGCCAGCTCAAACAT	ATCAAGAAGAGTAGTACCGCTAGCATTAC
qPCR*arp*<br>rplp0-201 ENSDART00000073462.5	CTGAACATCTCGCCCTTCTC	TAGCCGATCTGCAGACACAC

Supplementary Table 2. List of the primer sequences used for whole-mount *in situ* hybridization.

**Probe**	**Forward**	**Reverse**
zf*col6a1*	ATCGTGGACAGCTCTGAGAG	TCCGTGAGAAGTCCTTGGTC
zf*col6a2*	TGTGAAAAGAGATGCGGTGC	GATCCGGACAAAGCACAGTC
zf*col6a3*	AGGCTTTGCAAACCCGTAAG	TCCTCTAAGAAGCTGCGGAC

SUPPLEMENTARY FIGURES

Supplementary Figure S1. (**a**) Schematic diagram of the domain structure of ColVI α chains in zebrafish (*D. rerio*) and in mouse (*M. musculus*). The domain organization of the α1(VI), α2(VI) and α3(VI) chains (i.e., the main ColVI subunits) is largely conserved between fish and mammals, including humans. A distinctive feature of zebrafish and other cyprinid fish is the presence of two ohnolog genes coding for the α4(VI) chain (see also Fig. 1a, b). At difference from mouse α4(VI), the zebrafish α4(VI) chains display additional VWA modules at the N-terminal end (outlined in red in the diagram) and two Kunitz-like domains at the C-terminal end. Genes coding for the α5(VI) and α6(VI) chains are absent in zebrafish, suggesting that they evolved later during evolution. (**b**) Multiple amino acid sequence alignment of the α1(VI) chain in mouse (MmCol6a1), humans (HsCol6a1), zebrafish (DrCol6a1) and fugu (TrCol6a1), as determined by analysis with the ClustalW software. Identical amino acid residues in all sequences are marked with '*', while conservative and semi-conservative substitutions are marked with ':' and '.', respectively. The three VWA domains are highlighted in green, the collagenous domain triple helix is highlighted in red, and the conserved cysteine residues are boxed.

Supplementary Figure S2. (**a**) Phylogenetic analysis of zebrafish ColVI genes. Phylogenetic trees of the ColVI α chains of zebrafish (Dr), mouse (Mm) and human (Hs), obtained by amino acid sequence comparison of the regions spanning the C1 and C2 domains in the corresponding α chains. The sequences were aligned using the PILEUP program of the GCG package, using default parameters. The trees were constructed using the PROTEIN PARSIMONY, PROTEIN DISTANCE, FITCH and CONSENSE tools of the PHYLIP package version 3.695. Bootstrap analyses using 100 replicates were performed to show the significance. Numbers indicate the statistical weight of the individual branches. The N1 and N2 domains of human ColVI α3 chain were used as outgroup. (**b**) Comparative maps of syntenic regions of the zebrafish *col6a4a* gene. Genes encoding the corresponding α4 or the in tandem duplicated α5 and α6 chains in different species are indicated by red arrows. Neighbouring syntenic genes are indicated by blue arrows. For simplicity, the orientation of the genes on the chromosomes was adjusted to that of zebrafish chromosome 16. Note that the nomenclature for the genes encoding the long novel ColVI chains is not consistent for many species. Due to a large pericentric inversion on human chromosome 3, the gene coding for the α4 chain is split into two pseudogenes (*COL6A4P1* and *COL6A4P2*). Asterisks indicate genes that encode proteins containing at least parts of a Kunitz domain, a common feature for α4 chains.

Supplementary Figure S3. (**a**) Whole-mount *in situ* hybridization for *col6a2* and *col6a3* in zebrafish embryos and larvae. The panels show lateral (lat) and dorsal (dors) views of 1- to 3-dpf embryos labeled with *col6a2* or *col6a3* probes, as indicated. (**b**) Whole-mount *in situ* hybridization of a 24-hpf embryo labeled with the *col6a1* riboprobe. The inset shows higher magnification of the boxed area, revealing labeling at the level of basal epidermal cells (arrow). (**c**) Whole-mount immunofluorescence labeling with anti-ColVI in 2-dpf embryos injected with control (CTL MO) and exon 9 *col6a1* (Ex9 MO) morpholino oligonucleotides. Scale bar, 50 μm.

Supplementary Figure S4. Whole-mount immunofluorescence labeling with anti-ColVI (red) and anti-ColXII (green) antibodies in zebrafish larvae at different developmental stages from 2 dpf to 6 dpf, as indicated. The panels show lateral (lat), ventral (ven) and dorsal (dors) views of different head regions. From 2 dpf onwards, ColVI abundantly co-localize in the connective tissue surrounding the eye, olfactory pits and craniofacial cartilages. Nuclei were stained with Hoechst (blue). Scale bar, 50 μm. ch, ceratohyal; ey, eye; he, heart; mc, Meckel's cartilage; op, olfactory pit; pq, palatoquadrate.

Supplementary Figure S5. (**a**) Immunofluorescence with anti-ColVI antibodies in transverse sections of 6-dpf larvae at the level of the head, showing ColVI labeling in brain blood vessels, as also confirmed by the negative control (secondary antibody, II ab only), where ColVI labeling is absent. Nuclei were stained with Hoechst (blue). (**b-e**) Confocal z-stacks (ventral view) of whole-mount immunofluorescence for ColVI (red) and ColII (grey) in 3-dpf *Tg(7xTCF-Xla.Siam:GFP)ia4* (TCF:GFP, green) larvae. ColVI is present in the connective tissue surrounding craniofacial cartilages, in close proximity to Wnt responsive cells. (**f-i**) Confocal z-stacks of whole-mount immunofluorescence for ColVI (red) in 6-dpf *Tg(7xTCF-Xla.Siam:GFP)ia4* (TCF:GFP, green) larvae, showing ColVI labelling near Wnt-positive cells in jaw cartilages. Panel **i** is a higher magnification of an individual z-stack of panel **h** at the level of Meckel's and palatoquadrate cartilages. (**j-n**) Whole-mount immunostaining with ColVI (red) and ColXII (gray) antibodies and Hoechst (blue) in 6-dpf *Tg(osx:nuGFP)* (osx:GFP, green) larvae. ColVI and ColXII labelling surrounds dentary bone and operculum. (**o-s**) Confocal z-stack of whole mount immunostaining of 6-dpf *Tg(osx:nuGFP)* (osx:GFP, green) larvae operculum, stained with ColVI (red) and ColXII (gray) antibodies and Hoechst (blue). ColVI and ColXII are abundant in the operculum. (**t-x**) Whole mount immunostaining with ColVI (red) and ColXII (gray) antibodies and Hoechst (blue) in 2-dpf *Tg(fli1:EGFP)* (fli:EGFP, green), showing ColVI and ColXII deposition at the level of pharyngeal arches. Scale bar, 50 μm. branchiostegal ray; cb, ceratobranchial; ch, ceratohyal; db, dentary bone; ey, eye;; mc, Meckel's cartilage; ope, operculum; pa, pharyngeal arch; pq, palatoquadrate.

Supplementary Figure S6. (**a**) Immunofluorescence with anti-α1(VI) antibodies in transverse sections of 1-year old fish at the level of the trunk, showing the presence of strong ColVI labeling (red) in bony scales and dermis (left panel), in the endomysium of skeletal muscles (middle panel), and in the mucosa and muscular layers of gut (right panel). Nuclei were stained with Hoechst (blue). Scale bar 50 μm (c) or 25 μm (d). de, dermis; ep, epimysium; fb, fiber; me, muscularis externa; sc, scale; v, villus. (**b-q**) Immunofluorescence images of ColVI labeling in 5-mpf *Tg(7xTCF-Xla.Siam:GFP)ia4* zebrafish. Sections of 5-mpf *Tg(7xTCF-Xla.Siam:GFP)ia4* zebrafish were stained with anti-ColVI (red) and anti-GFP (green) antibodies. Nuclei were stained with Hoechst (blue). (**b-i**) Brain sections, showing ColVI labeling in blood vessels close to Wnt-responsive cells. (**j-q**) Section of basihyal cartilage. ColVI surrounds Wnt-responsive cells, as revealed by the magnification of the boxed areas in panels **n'-q'**. Scale bar, 50 μm (b-e) or 100 μm (f-m).

a

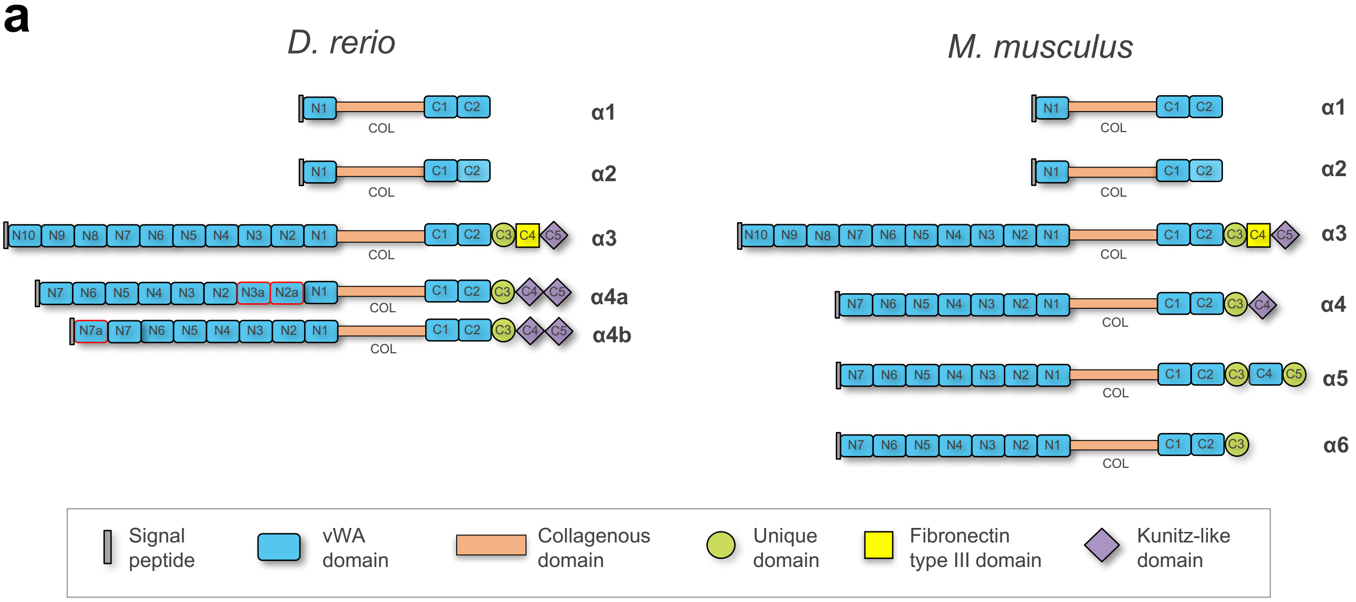

b

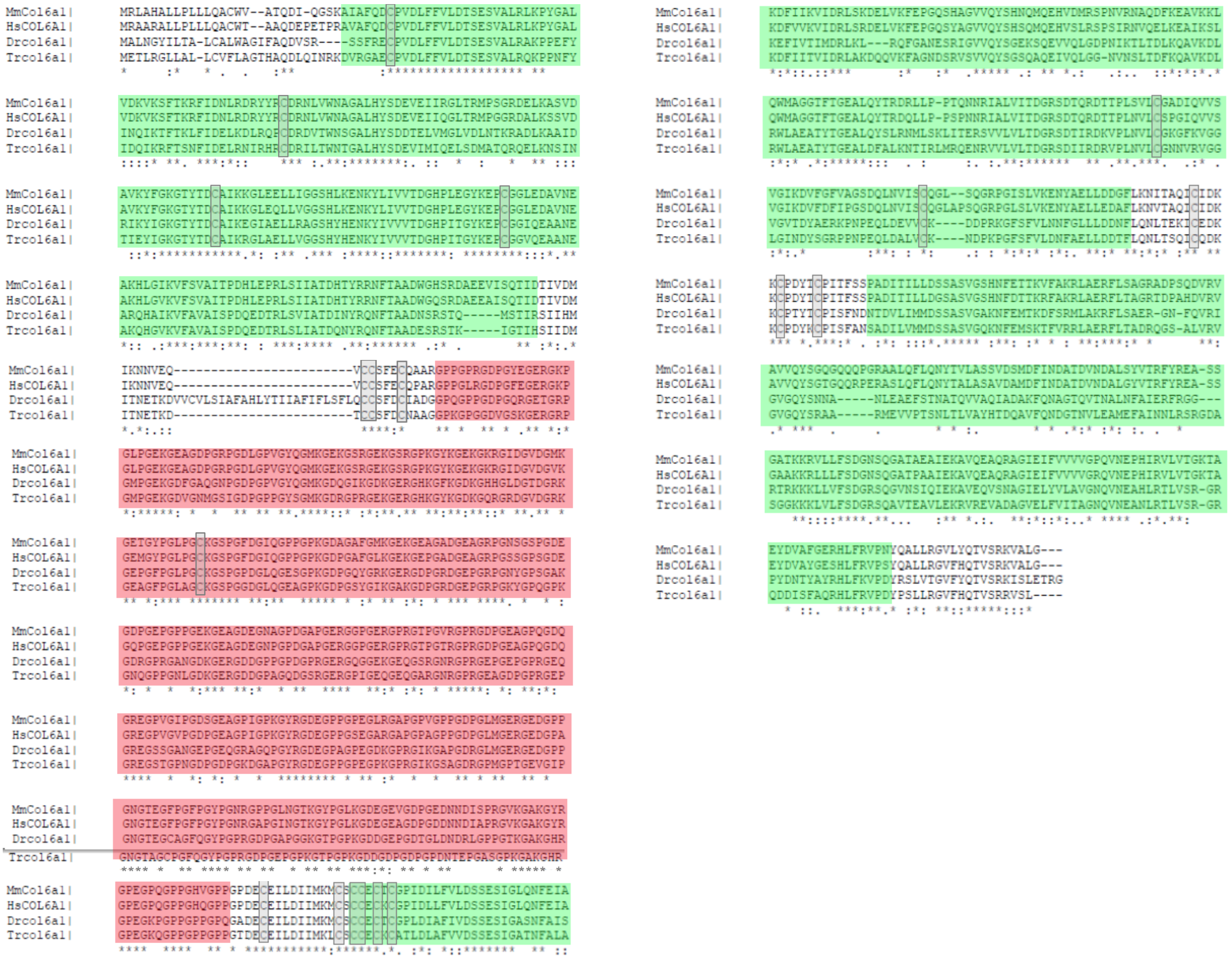

Supplementary Figure S1

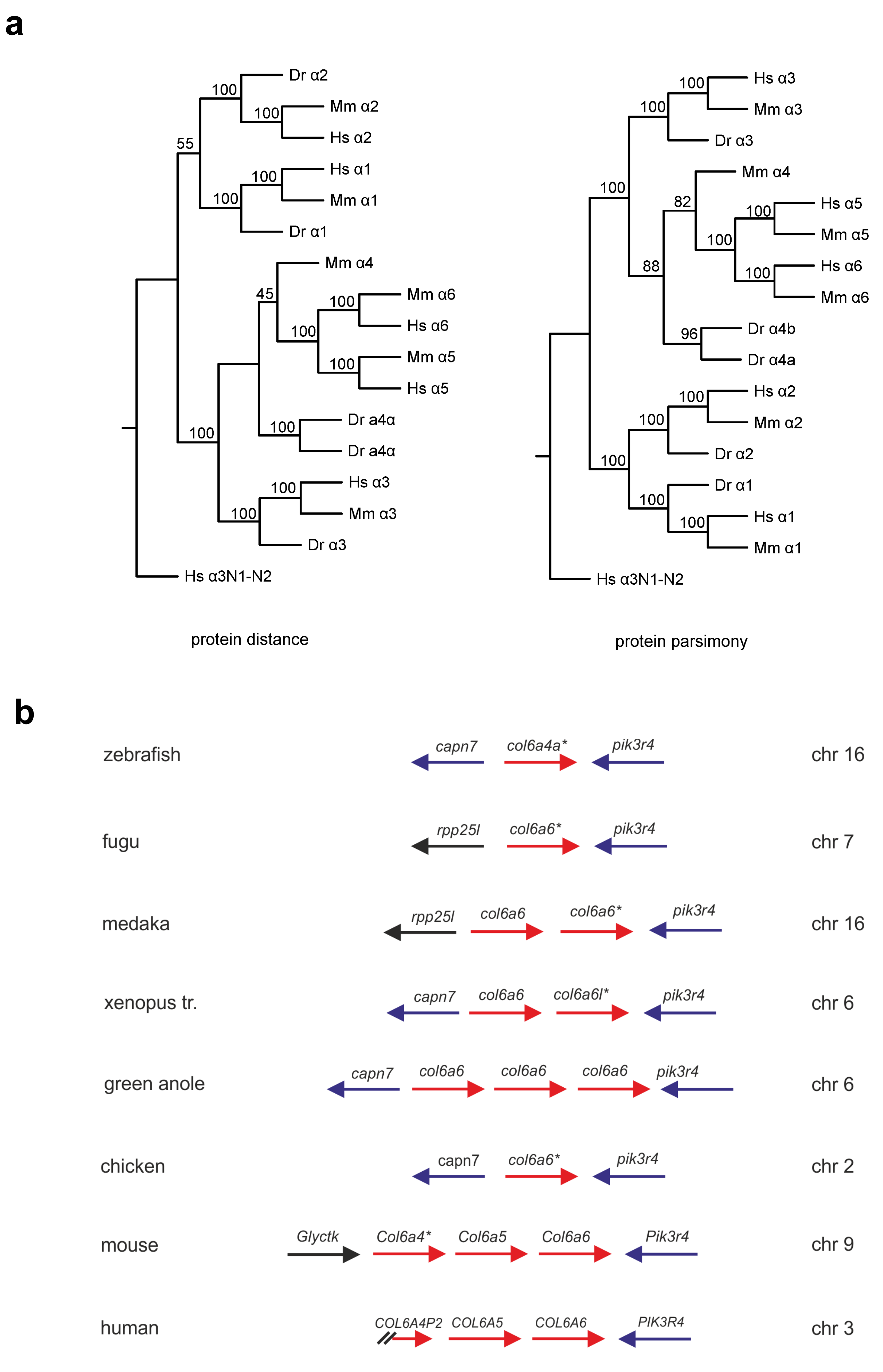

Supplementary Figure S2

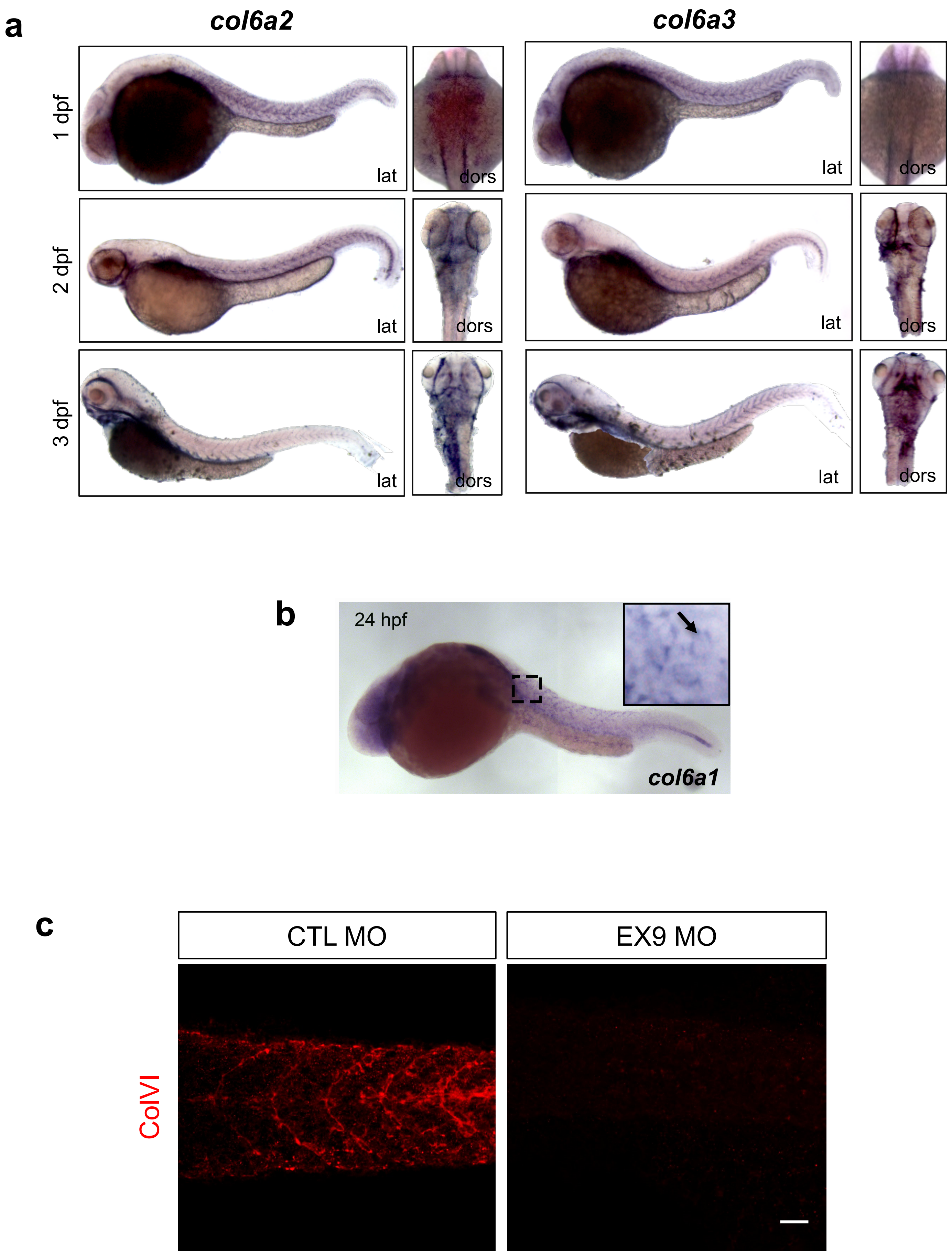

Supplementary Figure S3

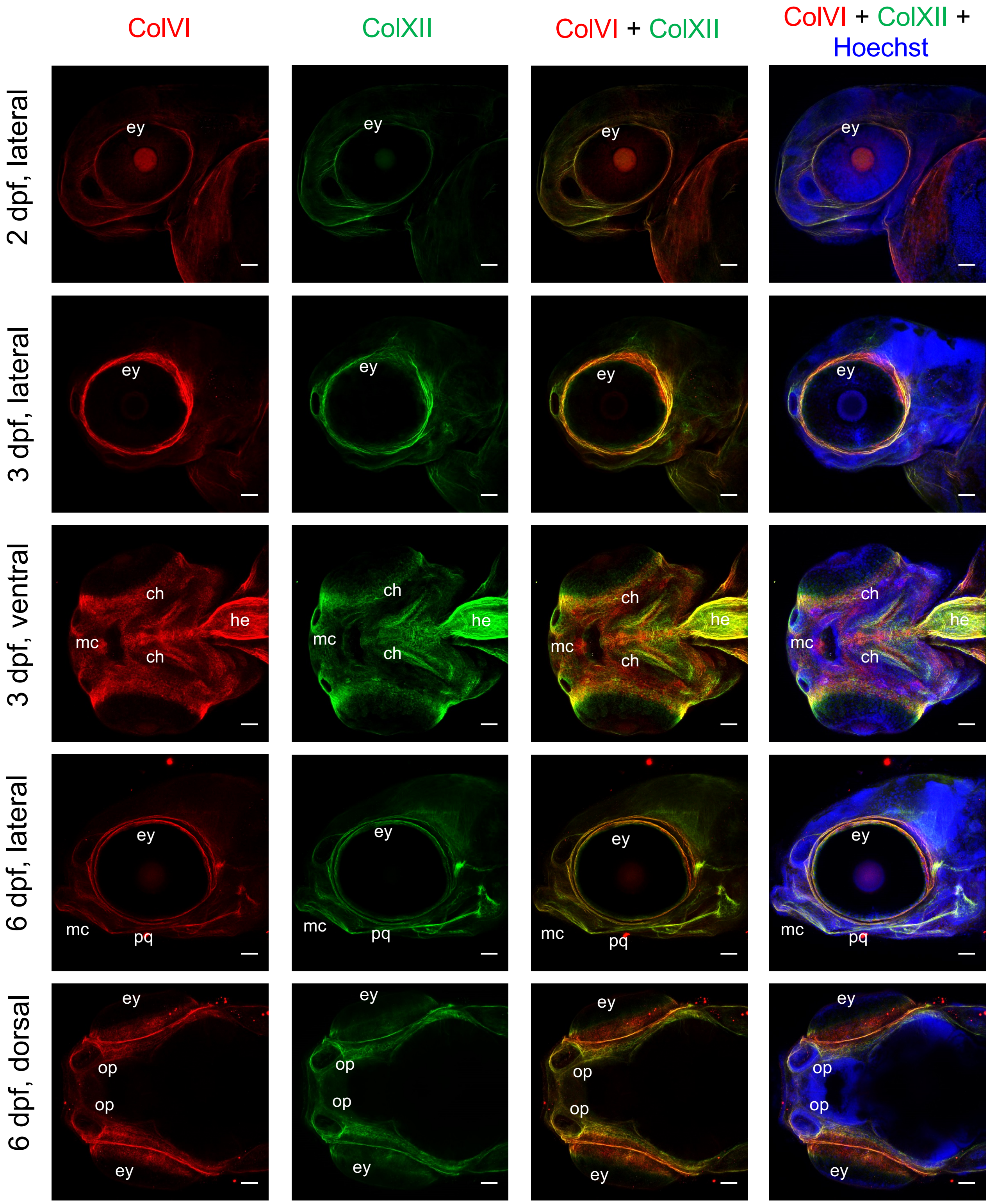

Supplementary Figure S4

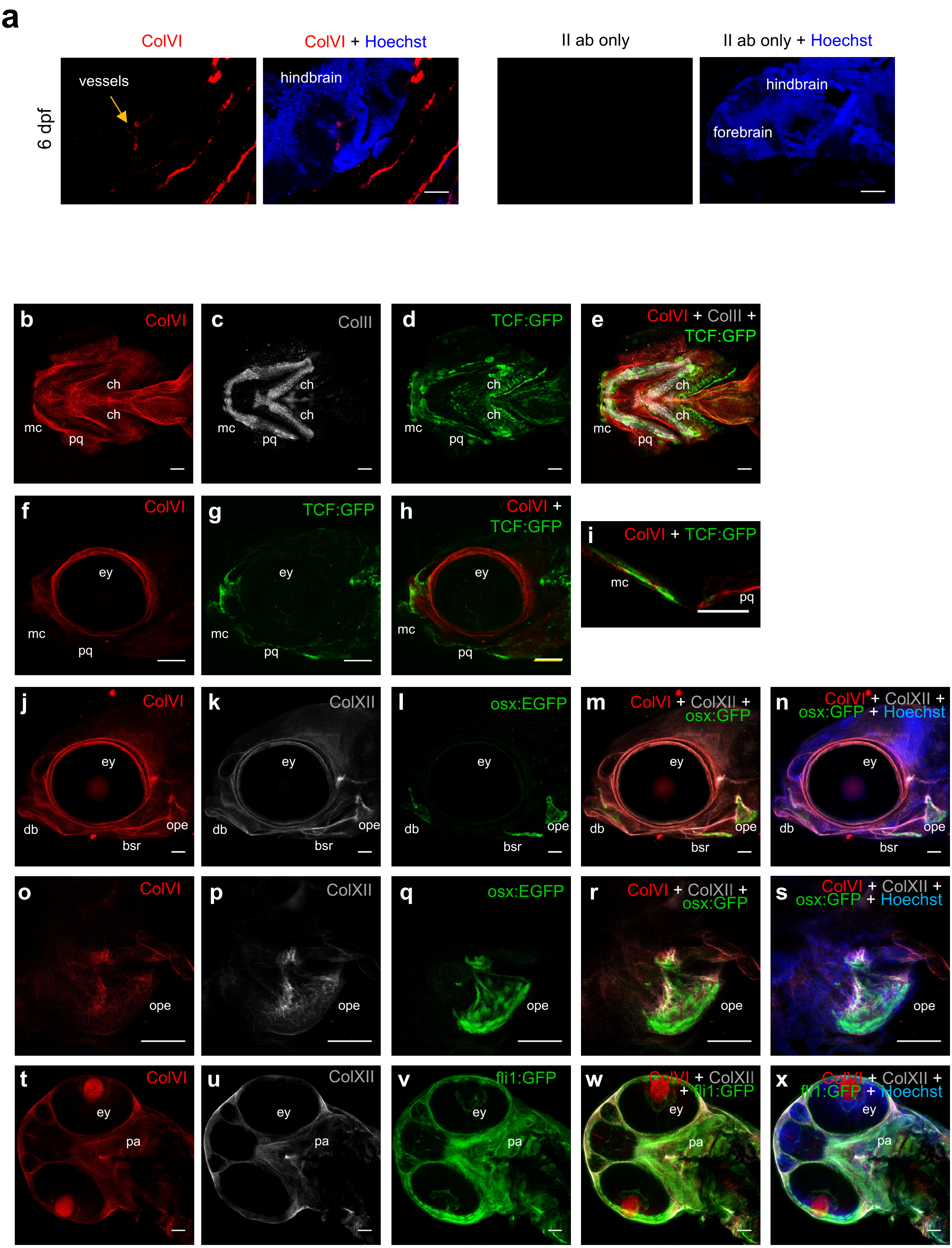

Supplementary Figure S5

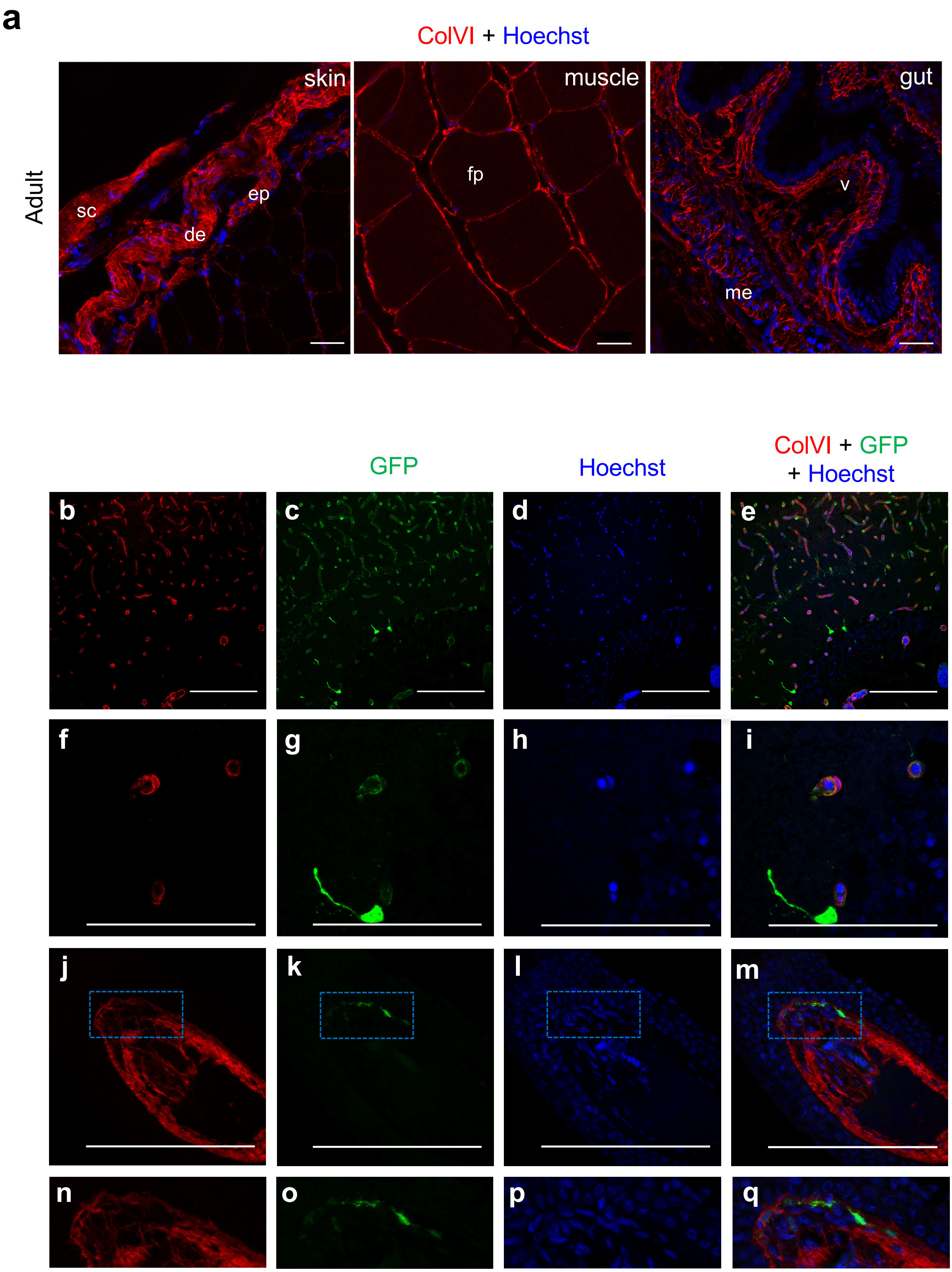

Supplementary Figure S6
